# Supplementary material for: Electromotive Enhanced Drug Administration in Oncology: Principles, Evidence, Current and Emerging Applications
Source: Cancers (Basel). 2022 Oct 11;14(20):4980. doi: 10.3390/cancers14204980 (PMC9599758; doi:10.3390/cancers14204980)
Supplement: Supplementary file 1 [file cancers-14-04980-s001.zip › cancers-1876576-supplementary.pdf]

**Supplementary material**

**File S1: Elaboration on the Fundamental Principles of EMDA**

The two main electrokinetic phenomena (electromigration and electroosmosis) in EMDA-mediated drug transport can be described by the modified Nernst-Planck equation [25]:

$$J_i = -D_i \left[\frac{\partial c_i}{\partial x} + \frac{F}{RT} z_i c_i \frac{\partial \phi}{\partial x}\right] + v c_i$$

Where $J_i$ denotes the flux density (mol*m$^{-2}$ s$^{-1}$) of the ion $i$ under study, $D_i$ the diffusion coefficient, $\frac{\partial c_i}{\partial x}$ the concentration gradient, F denotes Faraday's constant, R the universal gas constant, T the absolute temperature expressed in Kelvin, $Z_i$ the charge of ion, $C_i$ the concentration, $\frac{\partial \phi}{\partial x}$ the electric potential gradient, and $v$ the average velocity of the solvent flow. The negative sign in the equation arises from a convention: the flux density is positive when the direction of the transport coincides with increasing distance (the distance coordinate is zero on the electrode surface, positive in solution and negative in the electrode).

The first term of the equation denotes drug ionic transport by passive diffusion (i.e., Fick's first law of diffusion) [26], while the second and third terms denote electromigration and electro-osmotic phenomena respectively. The total flux density of ion $i$ being transported is therefore a sum of the three individual flux densities, namely those related to diffusion, migration, and convective flow or electro-osmosis.

$$J_i = J_i^{dif} + J_i^{mig} + J_i^{con}$$

It is noteworthy to mention that the contribution of the diffusion transport is often considered negligible which means that the flux density is mainly governed by the two latter terms.

When electroporation is considered, the equation may be modified to [27]:

$$\boldsymbol{J_i} = \varepsilon \left\{-\boldsymbol{H_i D_i}\left[\frac{\boldsymbol{dC_i}}{\boldsymbol{dx}} + \boldsymbol{D_i C_i}\left(\frac{|\boldsymbol{Z_i}|\boldsymbol{F}}{\boldsymbol{RT}}\right)\left(\frac{\boldsymbol{d}\Psi}{\boldsymbol{dx}}\right)\right] + \boldsymbol{W_i v C_i}\right\}$$

where $H_i$ is the hindrance factor of diffusion and electromigration transport, $W_i$ the hindrance factor for electroosmosis, and $\varepsilon$ the combined effective porosity of the membrane as a result of electroporation.
